# Persistence of Psittacine Bornavirus-4 Viral RNA Is Temperature Dependent in Aqueous Environments and Material Dependent in Non-Aqueous Environments

**DOI:** 10.3390/vetsci12111065

**Published:** 2025-11-06

**Authors:** Caitlin P. Mencio, Kelsey Williams, Donald J. Brightsmith, Sarah A. Hamer

**Affiliations:** Schubot Center for Avian Health, Department of Veterinary Pathobiology, College of Veterinary Medicine and Biomedical Sciences, Texas A&M University, College Station, TX 77843, USA

**Keywords:** bornaviridae, parrots, RNA, virus, temperature

## Abstract

**Simple Summary:**

Psittacine bornavirus-4 (PaBV-4) infection causes gastrointestinal disease in companion birds, especially parrots. The ways by which the virus spreads are not well understood, nor is its ability to persist in the environment. Our study aimed to detect this virus (using RNA as a proxy for infectious virus) in various environmental conditions that are common to aviaries and houses, as well as test if the virus was infectious in cell culture. We found that PaBV-4 viral RNA detection was time and temperature dependent in liquid with cooler samples showing longer persistence and slower degradation. The presence of viral RNA was found on metal and plastic surfaces for longer when compared to wood and cloth. We showed that liquid samples containing PaBV-4 were infectious in cell culture. Understanding viral persistence in the environment can inform avian husbandry for aviaries or pet owners to prevent viral spread, especially in mixed infection status populations.

**Abstract:**

Psittacine bornavirus type-4 (PaBV-4) causes proventricular dilatation disease and death among diverse birds, most notably caged parrots and related species, with no known cure or vaccine. Infected birds can shed virus in fecal matter, urine, and feather dander but it is unknown how well PaBV-4 survives outside of the host. This study focused on assessing the persistence of PaBV-4 in common environmental situations. The presence of viral RNA was examined in aqueous solutions at varying temperatures and recovery from typical avian husbandry materials (plastic, wood, metal, and cloth). Viral RNA persistence in aqueous samples was found to be 3 weeks at 37 °C, 2 months at 24 °C (room temperature), and 3 months at 4 °C. Viral RNA was also recovered from plastic and metal surfaces up to 72 h after inoculation. Also examined were disinfection protocols comparing coverage versus contact time for a reduction in viral RNA. Complete coverage by the disinfecting agent was more important for preventing recovery of viral RNA. Additionally, PaBV-4 RNA was transferable by paper towel. These results provide the first evidence of the robust nature of PaBV-4 in an aqueous environment and show that cleaning protocols need to be carefully curated to limit possible viral spread.

## 1. Introduction

Avian bornaviruses (ABVs) are a group of enveloped RNA viruses that affect a wide range of avian species, both in captivity and the wild. The psittacine type of ABV (PaBV) is a neurotropic virus and is the only known etiological agent of proventricular dilatation disease (PDD), also called avian ganglioneuritis. Clinical signs of infection predominately relate to the gastrointestinal tract or nervous system. These signs include crop stasis, constant regurgitation, maldigestion, weight loss, ataxia, seizures, stargazing and feather destruction behaviors among other possible symptoms [1]. There is currently neither a cure nor vaccine for PaBV infection. While many infected birds are asymptomatic and can live long healthy lives, others develop severe clinical symptoms that often lead to death. Understanding viral transmission is a key factor in managing outbreaks within populations; however, the transmission of PaBV is not well understood. Shed viral RNA has been identified in fecal matter, urine, and feather dander from infected birds [2,3], likely allowing for natural transmission via indirect contact, such as fecal-oral, aerosol, or open wounds [4].

PaBV RNA has been detected in wild birds, including dabbling ducks, cranes, gulls, sandpipers, sea eagles, buntings, and parrots [5,6]. Analysis of the genetic structure of PaBV has shown closely related bornavirus sequences in various avian species, which suggests horizontal, inter-species transmission may be occurring [7]. However, PaBV is primarily of concern in captive populations where birds are in close, continuous contact, which may facilitate viral spread [1]. Additionally, unlike their wild counterparts, the living environment for captive birds is a restricted area, often dependent upon humans for maintenance and cleaning. This makes disease outbreaks of greater concern to the captive population. Outbreaks in such populations can have a large impact economically and emotionally for the owners and caretakers of these captive birds. As such, understanding virus survival and infectivity among different environments is an important part of protecting birds from PaBV transmission.

The role of the abiotic environment in the transmission of ABVs to birds remains largely unexplored but is critical for understanding how to manage areas with infected birds. Further, both caged and wild birds often access areas with diverse substrates, including water, metal, wood, and plastic, so understanding variation in the environmental persistence of the virus on different substrates is useful for understanding transmission risk pathways. Finally, understanding what prolongs or inhibits viral persistence can also inform best practices for cleaning and disinfecting areas, cages, and enclosures.

The objectives of this study were to examine how common environmental conditions affect the detectability of RNA from psittacine bornavirus-4 (PaBV-4). Our hypothesis is that increased time and elevated temperature will be associated with reduced detectability of viral RNA and reduced viral infectivity in cell culture.

## 2. Methods and Materials

All virus work was conducted in a biosafety level 2 (BSL2) laboratory environment, and any method involving virus open to the air was conducted in a biological safety cabinet.

Detectability of PaBV-4 RNA over time was performed for both liquid and solid surfaces. Each experiment was run in triplicate. Early time points for testing were determined by assuming a reasonable amount of time for an object (toy, food/water bowl, etc.) or water source would be available to a captive bird without cleaning or disinfection in an aviary or home environment. Final time points were chosen to correspond to situations where the surface or water source was not as easily removed or disinfected, i.e., cages o perches or water sources such as small ponds. Surfaces selected for study were those commonly found in products used in avian husbandry, such as bird toys (plastic), perching (wood), and cages (metal), as well as the people they may interact with (cotton cloth).

### 2.1. Harvest of PaBV-4-Positive Media from DEF Cells

Virus containing supernatant was harvested from duck embryonic fibroblasts (DEFs) that had been infected with PaBV-4 as previously described [8]. To prepare uninfected DEFs, fertilized duck eggs (Metzer Farms, Gonzales, CA, USA) were purchased and incubated on a rotating platform at 37.5 °C with 60% humidity for 11–15 days. Eggs were candled to confirm the presence of embryos, sprayed with 70% ethanol, and chilled at 4 °C for 1 h to kill the embryo. Eggs were sprayed again with 70% ethanol and placed in a biological safety cabinet where the narrow end of the shell was removed with sterile scissors. Embryos were extracted, placed in a Petri dish, decapitated, and the viscera removed. Each embryo was placed in a 75 mL glass trypsinizing flask containing a magnetic stir bar and 25 mL Dulbecco’s Phosphate-Buffered Saline (DPBS), without Ca or Mg (Gibco, ThermoFisher, Waltham, MA, USA), and gently swirled by hand to remove red blood cells. The DPBS was decanted, and this process was repeated twice. After the second DPBS wash, 25 mL of 0.05% trypsin-EDTA (Gibco, ThermoFisher, Waltham, MA, USA) warmed to 37 °C was added to the trypsinizing flask. The flask was placed on a stir plate and stirred at room temperature for 6 min to make a cell suspension. The trypsin suspension was then decanted through 8 layers of sterile cheesecloth into a sterile beaker on ice containing Dulbecco’s Modified Eagle Medium (DMEM, Gibco, ThermoFisher, Waltham, MA, USA). This process was repeated thrice using the same beaker and cheesecloth. The cell suspension was transferred to a 50 mL conical tube and centrifuged for 10 min at ~1000× *g* at 10 °C. The cell pellet was resuspended in freezing media using approximately 3 mL of media per embryo. The suspension was placed in 1 mL aliquots in cryogenic vials and stored at −80 °C until needed. To begin the culture, a frozen vial was thawed rapidly in a 37 °C water bath. After thawing, the 1 mL from the cryogenic vial was added to 5 mL of DMEM containing 10% fetal bovine serum (FBS) (DMEM/FBS) in a 15 mL conical tube that was then centrifuged for 5 min at ~1000× *g*. The cell pellet was resuspended in ~1 mL of DMEM/FBS, and 500 μL was added to a T-75 flask containing 10 mL of DMEM/FBS and incubated at 37 °C with 5% CO_2_. Media in the flask was changed at 24 h after seeding.

Uninfected cells were grown in a T-75 flask until confluent. The flask was then infected with 0.2 mL of previously infected cells by adding the inoculum to 10 mL of cell culture media (DMEM/10%FBS/1× Penicillin–Streptomycin) present in the flask. Cells were allowed to incubate for 2 h. The media was then removed, discarded, and replaced. Cells were next allowed to grow for 4 d, after which they were passaged. A 0.2 mL aliquot from the 2 mL cell suspension prior to plating was assessed at each passage by RT-PCR to determine viral infection (details below) and compared with 0.2 mL of inoculum. Once a strong positive was obtained by PCR, determined by two factors—similar band intensity between the cell suspension sample and the positive control and a plateauing of band intensity between passages when testing equal amounts of cell suspension, cells were passaged twice more into T-75 flasks. Once confluent again, media were removed and stored to test for viral RNA. Cells were passaged to maintain infected cell population to allow for the harvesting of virus containing conditioned media for all subsequent experiments. All removed media were tested for the presence of PaBV-4 RNA by PCR as described below. The supernatant was then stored at −20 °C.

### 2.2. Viral Inoculation and Sampling for Assessment of Effects of Temperature, Surface Type, and Sampling Method

Equal volumes of viral-RNA-positive conditioned media were separated into different experiments. For all experiments, 200 μL of media was set aside as the zero timepoint/stock measurement. To test the effect of temperature and time on liquid samples containing viral RNA, 200 μL was placed into 3 separate microcentrifuge tubes for each of the following timepoints measured: 24 h, 48 h, 72 h, 1 week, 2 week, 3 week, 4 week, 5 week, 6 week, 8 week, 3 month, and 6 month. One tube from each timepoint was placed at each of 4 °C, 37 °C or room temperature (~24 °C). Upon reaching the desired duration at that temperature, tubes were transferred to −20 °C and stored for downstream processing. Another set of 5 tubes was also made to examine the effects of freezing and thawing to ensure that the storage method would not affect our results. Each tube was stored at −20 °C, with one tube serving as the control by only being frozen after initial aliquoting and thawed only when ready to harvest the RNA. The other four tubes were removed from the freezer and allowed to thaw at room temperature, after which they were placed back in the −20 °C freezer to re-freeze. This action was repeated with 1 tube going through this process once, another twice, another thrice, and the final tube going through 4 rounds of additional freeze and thaw.

**Figure 1:**
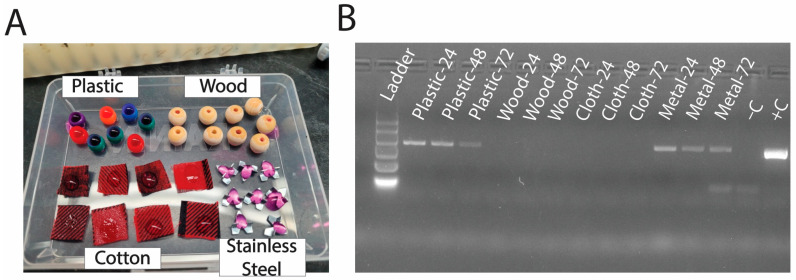
Detection of viral RNA from different substrates: (**A**) pictoral representation of plastic, metal, cotton cloth, and wood surfaces being incubated with PaBV-4-containing liquid; (**B**) PCR results from liquid used to wash each type of surface after 24, 48, and 72 h to determine the presence of viral RNA.

To test how long the viral RNA would persist on various surfaces, 200 µL of viral-RNA-positive supernatant was added to each of the following different surfaces that pet birds may commonly be in contact with through caging, perches, or enrichment, as follows: a plastic bead, a wooden bead, a piece of cotton cloth, and a piece of stainless-steel metal, as pictured in Figure 1A. One of each item was used for each timepoint (24 h, 48 h, and 72 h). Each item was in contact with the virus containing media on it for ~2 h, tapped once against a paper towel to remove excess liquid, then transferred to an empty well of a 24-well plate. The lid was placed on the plate and stored at room temperature. Virus retrieval was performed by pipetting 200 μL of sterile PBS over the area that had been exposed to PaBV containing media twice and then retrieving the PBS. These tubes were stored at −20 °C until processed for RNA extraction.

### 2.3. RNA Extraction and Conventional PCR Analysis

The RNA extraction was performed using the Quick-RNA viral kit (Zymo Research, Irvine, CA, USA) per the manufacturer’s instructions. Positive controls consisting of initial inoculum and negative controls consisting of sterile PBS were utilized for each PCR analysis run. First-strand cDNA was generated using the high-capacity cDNA reverse transcriptase kit with RNase inhibitor (Applied Biosystems, ThermoFisher, Waltham, MA, USA) using 10 μL RNA and random hexamers. A final volume of 20 μL was made for each reaction, which included 2 μL 10× buffer, 2 μL random hexamers, 0.8 μL 100 mM dNTP mix, 1 μL of RNase inhibitor, 1 μL reverse transcriptase, and PCR-grade water. A PCR primer set (forward primer: 5′-GGTAATTGTTCCTGGATGG-3′; reverse primer: 5′-ACACCAATGTTCCGAAGACG-3′) targeting a highly conserved portion of the matrix (M) protein found in avian bornavirus was used to screen for PaBV-4 [8]. PCR reactions performed using Platinum™ Taq DNA Polymerase High Fidelity (Invitrogen, ThermoFisher, Waltham, CA, USA). The PCR cycles were as follows: 94 °C for 2 min followed by 35 cycles of 94 °C for 30 s, 55 °C for 30 s, and 72 °C for 30 s, followed by a final extension at 72 °C for 5 min. PCR reaction products were run on a 2% agarose gel. Band intensity was compared between samples, as well as to the positive control, to assess the presence of viral RNA and as an index of viral RNA concentration [9].

### 2.4. Quantitative rt-PCR

Purified RNA from triplicate samples of ABV-positive media that was kept at 4 °C for 1 month, 3 months, 6 months, and 8 months; room temperature (~22 °C) for 1 week, 1 month, 2 months, and 6 months; and 37 °C for 1 week, 2 weeks, and 1 month were screened using qRT-PCR to determine the persistence of PaBV-4, as indicated by the recovery of viral RNA in the media. Purified RNA from ABV-positive and -negative media stock served as controls. This qRT-PCR assay was performed as previously described [10] and targets the P gene of PaBV. qScript XLT one-step RT-qPCR ToughMix (Quantabio, Louisville, KY, USA) was used to carry out first-strand cDNA and PCR amplification in a single reaction. The primers and probes (ThermoFisher Scientific, Waltham, MA, USA) were as follows:

ABV Fprimer: 5′-AAGAAGAA[Y]CC[Y]TCCATGATCTC-3′;
ABV Rprimer: 5′-AA[Y]TGCCGAAT[B]A[R]GTCATC-3′;
ABV Probe: 5′-FAM-TCGATAACTG [Y]TCCCTTCCGGTC-3′-QSY.

QPCR was carried out using the CFX96 Real-Time System with a C1000 Touch™ Thermal Cycler (BioRad, Hercules, CA, USA). The protocol was as follows: 1 cycle of 5 min at 50 °C (reverse transcription) followed by 1 cycle of 1 min at 95 °C (initial denaturation) and then 45 cycles of 3 s at 95 °C (denaturation) and 30 s at 60 °C (amplification and data collection). The relative change in gene expression was determined by examining shifts in the ct values in the samples collected over time and comparing them to the ct value of the positive and negative controls.

### 2.5. Disinfection Protocols

A total of 2 mL of ABV-positive conditioned media was placed into 28 60 mm plastic Petri dishes (Corning, Corning, NY, USA) and allowed to incubate at room temperature for 2 h. Media were removed by pipette, and dishes were divided into experimental groups of 8 dishes each for 70% ethanol, Bacdown™ (2.25% n-alkyl dimethyl benzyl ammonium chlorides, 2.25% ammonium chlorides, and 95.5% inert ingredients, Decon Labs, Inc., King of Prussia, PA, USA), or Rescue™ (accelerated hydrogen peroxide, Virox Animal Health, Oakville, ON, Canada), and 3 dishes for examining viral transfer by paper towel. The final dish was used as the positive control for viral RNA recovery. Each dish was placed in the center of a 6-inch-square marked area of a flat surface in a biological safety cabinet. This area was then sprayed once or five times using either a standard spray bottle, in the case of the diluted bleach, alcohol, and Bacdown™ solutions, or the manufacturer’s bottle, in the case of Rescue™, from a distance of approximately 8–10 inches away. Dishes receiving a single spray were allowed to sit for 5 m while dishes receiving 5 sprays had contact time of 1 min or 5 min. For each disinfectant, 3 dishes received single sprays, 2 dishes received 5 sprays/1 min, and 3 dishes received 5 sprays/5 min, for a total of 8 dishes per group. Petri dishes were inverted at the end of the time to remove any excess or pooled liquid that may have resulted from the disinfectant sprays. After inversion, the surface of the dish was swabbed thrice with sterile, polyester-tipped swabs by running the swab from one edge to the other in a straight line from three different starting points. The swab was then placed in 200 μL of sterile PBS.

To simulate some wiping steps with cloth that may commonly be used in cleaning, 3 dishes received a single spray of 70% ethanol as previously described. Following the 5 min of contact time, each dish was wiped with a paper towel. The same paper towel was then used to wipe 3 successive clean Petri dishes. The surfaces of these dishes were then swabbed as previously described and the cotton tip of the swab was placed into 200 µL of sterile PBS.

### 2.6. In Vitro Infection of DEF Cells

DEF cells were grown to confluency and then seeded into the wells of a 24-well plate at a density of 5 × 10^4^ cells/well. Cells were allowed to reach ~80% confluency at which time the media were replaced with 250 μL of DMEM containing penicillin/streptomycin, 10% fetal bovine serum, and 50 μL of testing sample. Cells were incubated overnight at 37 °C with 5% CO_2_ and then the media were replaced with 500 μL of fresh media. Cells were allowed to continue to grow for 48 h at which time DEF cells were fixed for 12 min using 4% paraformaldehyde (PFA). After fixing, cells were stained as previously described [8]. In short, cells were washed with DPBS without Ca or Mg twice for 5 m and then permeabilized in 1% Triton X-100/DPBS for 10 min. Cells were then rinsed twice in DPBS without Ca or Mg for 5 min with gentle rocking. Next, cells were blocked in 5% skim milk in PBS containing 0.05% Tween-20 (PBST) at room temperature for 30 min. Cells were incubated in primary antibody solution (serum from a PaBV-positive cockatiel–PaBV infection and antibodies confirmed by both PCR and ELISA, 1:250, in blocking solution) overnight at 4 °C. Cells were then washed by gently rocking for 5 min thrice in PBST. Cells were incubated in secondary antibody solution, AP-conjugated goat anti-macaw antibody (Bethyl Laboratories, Montgomery, TX, USA owned by Fortis Life Sciences, Waltham, MA, USA), 1:5000, in PBST and incubated for 1 h at room temperature. Cells were washed thrice with PBST for 5 min. Cells were then rinsed with ultrapure water and 200 μL of BCIP/NBT (5-bromo-4-chloro-3-indolyl phosphate/nitro blue tetrazolium), liquid substrate for alkaline phosphatase, was added to each well. Virus-infected cells appeared as purple foci and were imaged using a 10× objective on an Olympus microscope.

### 2.7. Statistical Analysis

One-tailed T-tests were performed to compare the distribution of CT values between the 0 time point and the latest time point for which viral RNA was detected (3 months, 2 months, and 2 weeks for the 4 °C, room-temperature, and 37 °C experiments, respectively). All other data were analyzed descriptively without inferential statistics

## 3. Results

### 3.1. PaBV-4 Viral RNA Can Be Detected in Liquid for over 8 Weeks

**Figure 2:**
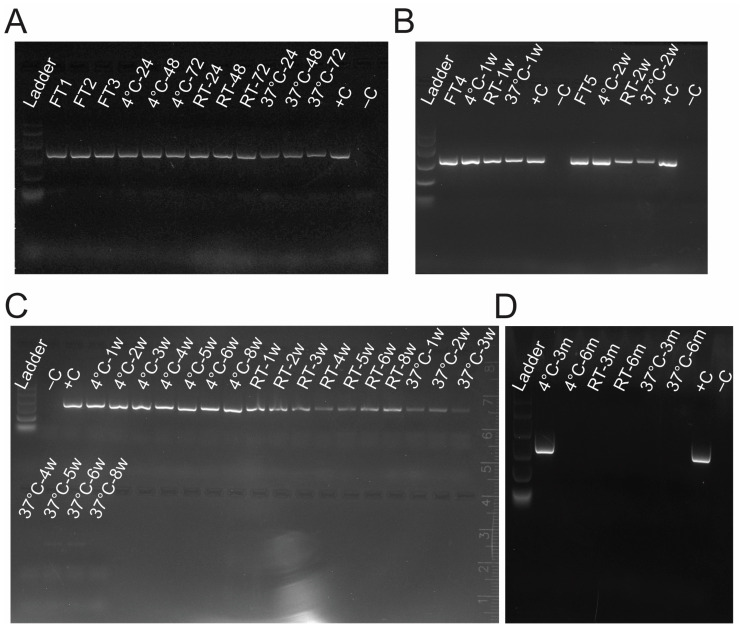
PaBV-4 persists in liquid for 3+ weeks depending on the temperature: (**A**) PCR results from PaBV-4-containing conditioned media that were subjected to up to three freeze–thaw cycles (FT) or stored at either 4 °C, room temperature (RT), or 37 °C for 24, 48, or 72 h; (**B**) PCR amplification of viral RNA from a 4th and 5th round of FT and storage at all three temperatures for 1 or 2 weeks (w); (**C**) PCR results for the presence of PaBV-4 viral RNA in liquid samples stored at all three temperatures for up to 2 months (m); (**D**) PCR results for the presence of PaBV-4 viral RNA in liquid samples stored at all three temperatures from 3 to 6 m.

Viral RNA was present after each of the five rounds of the freeze–thaw cycle (Figure 2A,B), with no observable decrease in gel band intensity between cycles. Storage temperature showed an effect on the detectability of viral RNA. Samples kept at 37 °C showed an initial loss of intensity in the PCR band (Figure 2A). However, following that initial reduction the band remained stable for 2 weeks (Figure 2C). After 2 weeks, samples kept at 37 °C showed a quick degradation, with the PCR band disappearing after 1 month of storage (Figure 2C). Samples kept at room temperature showed a decrease in band intensity beginning at 2 weeks with another drop at around 1 month (Figure 2B,C). Viral RNA was detected in media stored at room temperature at 8 weeks but was gone by the 3-month time point (Figure 2C,D). PaBV-4-conditioned media kept at 4 °C was positive for viral RNA up to 3 months but was gone by the 6-month mark (Figure 2A–D).

**Figure 3:**
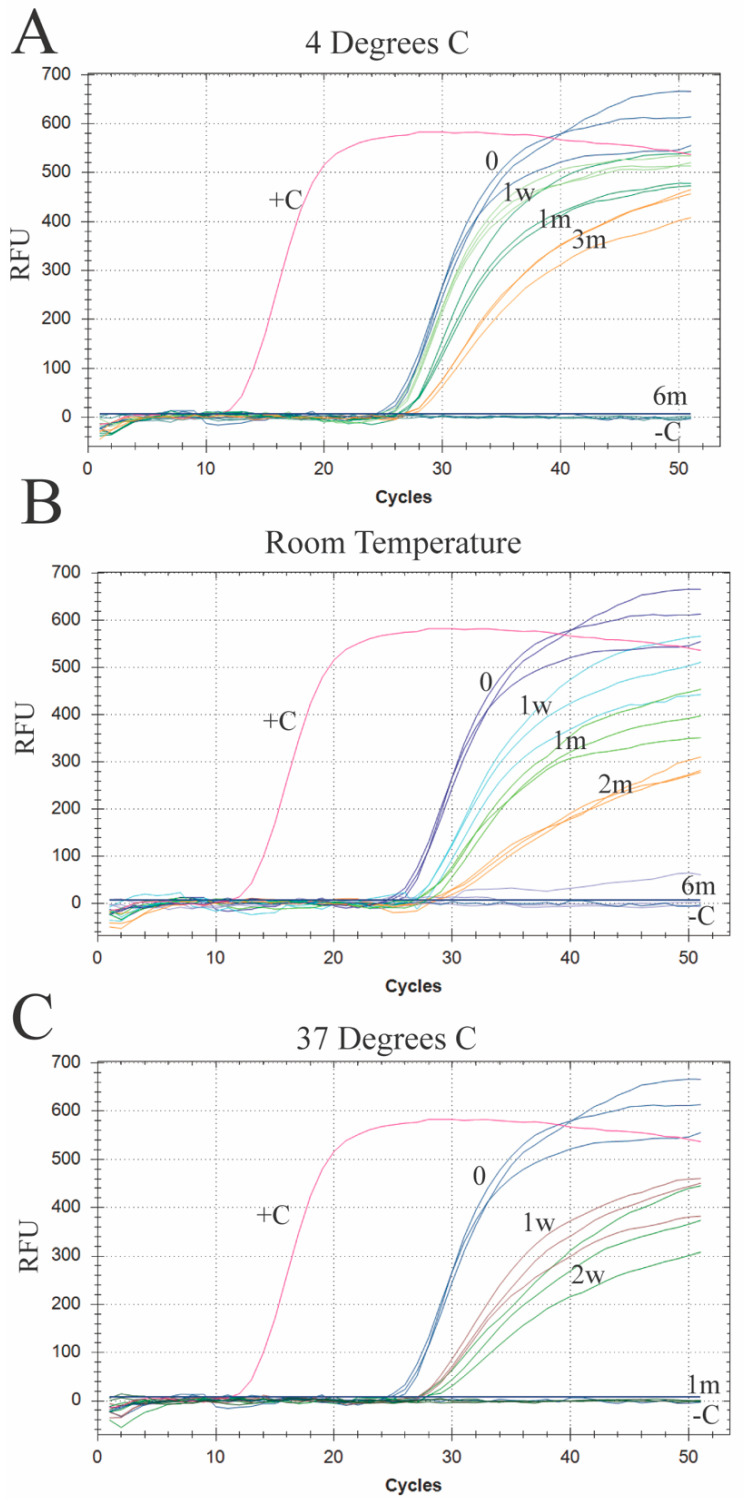
RNA recovery over time at different temperatures in aqueous solutions: (**A**) qPCR graph of a sample taken from PaBV-4 solution stored at 4 °C for a period of 0 h, 1 week (w), 1 month (m), 3 m, and 6 m along with a positive (+C) and negative (–C) control. Each color represents a single time point run in triplicate. (**B**) qPCR graph of a sample taken from PaBV-4 solution stored at room temperature for a period of 0 h, 1 w, 1 m, 2 m, and 6 m along with a positive (+C) and negative (–C) control; (**C**) qPCR graph of a sample taken from PaBV-4 solution stored at 37 °C for a period of 0 h, 1 w, 2 w, and 1 m along with a positive (+C) and negative (–C) control.

RT-qPCR analysis of the samples (*n* = 3) showed a slight increase in ct values compared to the 0 h control for samples at 1 month, 3 months, and 6 months at 4 °C (27.02 ± 0.20 to 27.37 ± 0.20, 28.42 ± 0.10 and 29.61 ± 0.25, respectively). The increase in ct values was significant between 0 h control and 6 months (*p* < 0.002). Samples taken at 8 months were akin to negative control samples, showing no viral RNA (Figure 3A). RT-qPCR of the samples at room temperature (*n* = 3) showed increased ct values compared to 0 h control (27.02 ± 0.20) at 1 w (28.73 ± 0.37), 1 month (29.77 ± 0.32), and 2 month (32.12 ± 0.41), while the 6-month sample was similar to the negative control (Figure 3B). The increase in ct values was significant between 0 h control and 6 months (*p* < 0.003). Samples (*n* = 3) taken at 1 week (29.60 ± 0.39) and 2 weeks (30.66 ± 0.78) from stock solution stored at 37 °C showed higher ct values compared to the 0 h control (27.02 ± 0.20), with samples taken at 1 month showing no viral RNA present, similar to the negative control (Figure 3C). The increase in ct values was significant between 0 h control and 1 months (*p* < 0.011).

### 3.2. Viral RNA Can Be Detected on Plastic and Metal Surfaces 72 h After Contamination

The PaBV-4 RNA was detected on plastic and metal surfaces at 24, 48, and 72 h post-inoculation (Figure 1B). The band intensity for both plastic and metal samples decreased over time, implying a decrease in viral RNA. In contrast, no viral RNA was recovered from the cloth or wooden samples at any time points (Figure 1B).

### 3.3. Virus Recovered from Surfaces and Liquid Is Infectious in Cell Culture

**Figure 4:**
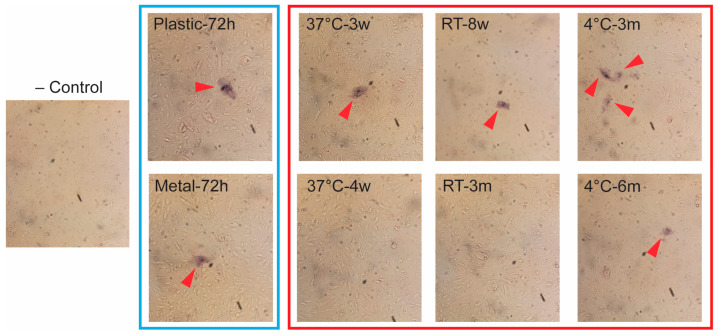
Testing infectivity of recovered virus from substrates and aqueous solutions in DEFs. Treatment of DEF cells with virus recovered from the last PCR-positive and first negative (if applicable) time point for plastic and metal (framed in blue) and from PaBV-4-containing liquid samples stored at different temperatures (framed in red). Red arrowheads indicate cells containing viral proteins as determined by BCIP/NBT reaction with alkaline phosphatase found on secondary antibody.

Uninfected DEFs showed no staining. However, DEFs that had been treated with recovered virus from both plastic and metal at 72 h were infected (Figure 4). In terms of PaBV-4-positive media stored at different temperatures, all samples showed the ability for infection at all time points for which a positive PCR result was observed, as follows: 3 months for 4 °C, 8 weeks for room temperature, and 3 weeks for 37 °C (Figure 4). In addition, infection was observed in cells treated with PaBV-4-contaminated media stored at 4 °C for 6 months, even though PCR testing of these samples indicated no viral RNA. Treatment of cells with 2-month-old RT samples and 1-month-old 37 °C samples, which were also PCR-negative, showed no sign of infection.

### 3.4. Coverage Is More Important than Contact Time or Disinfectant Type for Disinfecting PaBV-4 Viral Contamination

**Figure 5:**
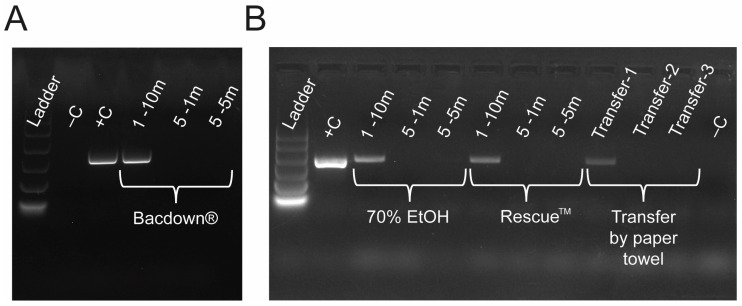
Coverage of contaminated surface is critical to elimination of viral RNA. (**A**) 1 or 5 sprays of BacDown^®^ with differing contact times. Recovery of virus drops with more coverage regardless of contact time duration. (**B**) Recovery of viral RNA after 1 or 5 sprays of 70% ethanol (EtOH) and Rescue^TM^ (AHP), showing that the coverage is more effective at eliminating viral RNA. Also, when left untreated, viral RNA can be transferred from one surface to another via paper towel at least once.

In all instances where five successive sprays of cleaning agent (70% ethanol, Bacdown™ and Rescue™) were employed, no viral RNA was detected by PCR (Figure 5A,B), indicating that all three disinfectants degraded PaBV-4 RNA. This observation was true regardless of whether it had 1 min or 5 min of contact time, indicating that liberal application of cleaning agents resulted in rapid degradation of viral RNA. By comparison, with a single spray of disinfectant on a contaminated surface, the 5 min contact time did not remove all viral RNA. Compared to the inoculum-positive control, the band intensity for samples recovered following a single spray of cleaner was reduced under every condition (Figure 5A,B). Additionally, using a paper towel to dry the dish after a single spray of 70% ethanol allowed for the transfer of viral RNA from the original surface to another Petri dish (Figure 5B). Attempts to continue to transfer viral RNA to tertiary and quaternary surfaces did not result in positive PCR results (Figure 5B).

## 4. Discussion

This study focuses primarily on RNA recovery as an indirect indicator of viral persistence [11,12]. Using this assessment of RNA, we explored the ability of PaBV-4 to survive under different environmental conditions. PaBV-4 persists in liquid media with the ability to infect cells after months of storage at a range of temperatures. However, it is more vulnerable to degradation at higher temperatures. The virus can persist for at least 3 days on two common materials, plastic and metal, that commonly encounter avian feces in bird cages. Proper cleaning methods that fully cover and saturate contaminated areas with cleaning solutions are needed to remove PaBV-4 from surfaces. This is even more important as improper cleaning can result in spreading the virus from one surface to another, as evidenced by the paper towel transfer experiment.

The finding that viral RNA can remain present for several months in liquids has potential implications for flock management. Although the survival of viruses tends to be higher in sterile water [13,14], our data show that detection of PaBV-4 virus in conditioned media, which contains cellular debris and fetal bovine serum, over time is quite robust, especially at lower temperatures. Although some viral degradation was observed via qPCR, the persistence was fairly robust at 4 °C into the 3rd month, showing only a 2 ct shift in qPCR values. This was less robust as the temperature increased, with loss of viral RNA occurring between 2 and 6 months at room temperature and between 2 weeks and 1 month when PaBV-4 is stored at 37 °C. This implies that heat has a detrimental effect on the detectability of the viral RNA, which is in line with similar RNA viruses. In regards to the implications for husbandry, since PaBV-positive birds have a highly variable disease progression following infection, it is not uncommon for birds to be asymptomatic while actively shedding the virus [1,2]. Shared water sources for captive or wild birds could be a place for transmission, as shedding by asymptomatic birds could potentially contaminate water sources for months depending on the ambient temperature. Further examination of virus infectiousness in different liquid mediums, as well as testing of water sources used by infected birds, may provide additional information about the likelihood of viral persistence across a wider range of environmental conditions. Such a transmission mechanism is similar to avian influenza which can also be transmitted via the abiotic environment [15,16]. Although transmission of ABV is not as well understood as influenza, many of the commonly proposed mechanisms require a similar introduction of the virus to the mucus membranes or open wounds. The ability of PaBV-4 to remain present and infectious in aqueous environments could facilitate these forms of viral transmission.

We found that viral RNA was detected on plastic and metal but not wood or cloth. Both wood and cloth absorb liquids, likely dispersing them over a larger area allowing for faster drying times. However, it is important to note that this study did not test thicker absorbent material, such as sponges or foam. A thicker material, especially one that is designed to retain liquid instead of drying out so quickly, may allow for virus recovery. Plastic and metal are non-absorbent allowing for a possible pooling of the liquid, which would allow for the maintenance of a damp environment for longer periods of time. This may allow for a higher concentration of virus or other aspects of the conditioned media in a single place, which could have some benefits for viral RNA detectability and/or viral survival. These data underscore the importance of proper cleaning procedures for reducing viral transmission.

The use of cleaning agents is a common method for eliminating transmissible viruses and containing a viral outbreak. However, these agents can be applied in a variety of ways, and some of these methods may be less efficient at eliminating virus. The implication from our results is that the type of cleaning agent, as long as it is virucidal, is less important than ensuring effective coverage of the contaminated area. However, only a relatively small subset of cleaning agents and relatively short contact times were utilized in this study. It is possible that other disinfectants may not be as efficient. Additionally, a strictly observed contact time is not common in the average cleaning routine, and one minute may be longer than most people would wait before wiping a surface down. Further studies should examine the efficacy of applications under a minute, as well as look at different types of disinfecting methods such as disinfecting wipes.

This study does have some clear limitations. Quantification with viral titers was not performed which limits our ability to make a clear determination of the decay rates of infectious virus versus recovery of viral RNA. Additionally, we only tested one specific strain, PaBV-4, of avian bornavirus. Other strains of pscitticine or avian bornavirus may be more or less susceptible to the same environmental conditions. Finally, only the viral samples recovered from liquid was tested in cell culture, as such while we showed the recovery of viral RNA on common household surfaces decreased over time, we did not show if this virus was capable of infecting cells.

This study serves as one of the first steps towards understanding the survival of ABVs in the environment. By examining the detectability of viral RNA in liquid, as well as on common surfaces, this study provides data that demonstrate that ABVs remain detectable in avian-friendly environments for days if not weeks, implicating an environmental role in possible horizontal transmission. Determining the environmental factors that assist in maintaining levels of virus provides valuable information that can be used to better elucidate the transmission of ABV, as well as assist in the reduction of viral spread.

## Data Availability

qPCR data presented in the study are openly available in the OAKTrust Digital Repository at https://hdl.handle.net/1969.1/1595891 (accessed on 25 September 2025). All other original contributions present in this study are included in the article. Further inquiries can be directed to the corresponding author.
